# Identification of multiple cancer-associated myositis-specific autoantibodies in idiopathic inflammatory myopathies: a large longitudinal cohort study

**DOI:** 10.1186/s13075-017-1469-8

**Published:** 2017-11-25

**Authors:** Hanbo Yang, Qinglin Peng, Liguo Yin, Shanshan Li, Jingli Shi, Yamei Zhang, Xin Lu, Xiaoming Shu, Sigong Zhang, Guochun Wang

**Affiliations:** 1 Department of Rheumatology, Beijing Key Lab for Immune-Mediated Inflammatory Diseases, China-Japan Friendship Hospital, 2 Yinhua Road, Chaoyang District, Beijing, 100029 China; 2 Graduate School of Peking Union Medical College, Beijing, 100730 China; 3 Department of Rheumatology, Lanzhou University Second Hospital, Gansu province, 730046 China

**Keywords:** Dermatomyositis, Polymyositis, Immune-mediated necrotizing myopathy, Myositis-specific autoantibodies, Cancer

## Abstract

**Background:**

Cancer is a significant complication contributing to increased mortality in idiopathic inflammatory myopathies (IIMs), and the association between IIMs and cancer has been extensively reported. Myositis-specific autoantibodies (MSAs) can help to stratify patients into more homogeneous groups and may be used as a biomarker for cancer-associated myositis. In this study, we aimed to systematically define the cancer-associated MSAs in IIMs.

**Methods:**

Serum from 627 patients with IIMs was tested for MSAs. The cancer risk with different MSAs was estimated by standardized incidence ratio (SIR). Paraneoplastic manifestation, such as the close temporal relationship between myositis onset and cancer diagnoses in patients with different MSAs, was also evaluated.

**Results:**

Compared with the general Chinese population, patients with IIMs and anti-transcriptional intermediary factor (TIF1)-γ antibodies (SIR = 17.28, 95% CI 11.94 to 24.14), anti-nuclear matrix protein (NXP2) antibodies (SIR = 8.14, 95% CI 1.63 to 23.86), or anti-SAE1 antibodies (SIR = 12.92, 95% CI 3.23 to 32.94), or who were MSAs-negative (SIR = 3.99, 95% CI 1.96 to 7.14) faced increased risk of cancer. There was no association between specific MSAs subtypes and certain types of cancer. Paraneoplastic manifestations were observed in the patients carrying anti-TIF1-γ, as well as other MSAs. There were no prognostic differences among the patients with cancer-associated myositis (CAM) from different MSAs subgroups. However, in comparison to those with cancer unrelated to myositis, CAM had a worse prognosis, with an age-adjusted and sex-adjusted Cox hazard ratio (HR) of 10.8 (95% CI 1.38-84.5, *p* = 0.02) for all-cause mortality.

**Conclusions:**

Our study demonstrates in what is, to our knowledge, the largest population examined to date, that anti-SAE1, and previously reported anti-TIF1-γ and anti-NXP2 antibodies, are all associated with an increased risk of cancer in patients with IIMs. Moreover, our data suggest that in some cases, anti-HMGCR, anti-Jo-1 and anti-PL-12 antibody production might also be driven by malignancy. This can aid in the etiologic research of paraneoplastic myositis and clinical management.

**Electronic supplementary material:**

The online version of this article (doi:10.1186/s13075-017-1469-8) contains supplementary material, which is available to authorized users.

## Background

Idiopathic inflammatory myopathies (IIMs) are a group of acquired, heterogeneous, systemic diseases that mainly affect skeletal muscle, of which polymyositis (PM), dermatomyositis (DM), immune-mediated necrotizing myopathy (IMNM) and sporadic inclusion body myositis (sIBM) are the main clinical subtypes [1]. Cancer-associated myositis (CAM) has a worse prognosis in afflicted patients, thus, clarifying whether specific phenotypic characteristics are associated with an increased risk of cancer in IIMs may help identify patients who could benefit from targeted malignancy screening.

The conflicting findings on the association between increased cancer risk and IIM subtypes may possibly be due to the significant clinical heterogeneity of patients with IIMs. Interestingly, myositis-specific autoantibodies (MSAs) are found exclusively in IIMs and can help to stratify patients into more homogeneous groups [2]. It is well-known that adult patients with dermatomyositis (DM) who carry anti-transcriptional intermediary factor (TIF1)-γ are more likely to develop malignancy than anti-TIF1-negative patients. Besides anti-TIF1-γ antibodies, a few studies have indicated that the presence of other MSAs, including anti-nuclear matrix protein (NXP2), anti-Jo-1, anti-Mi-2β, anti-HMGCR, is correlated with cancer in IIMs as well [3–12], but these findings have not been consistently replicated in other studies [13–16].

While the exact pathogenesis of CAM remains unclear, a paraneoplastic nature was assumed in the majority of these patients because cancer diagnosis and myositis onset seemed to temporally coincide. Additionally, improvement in myositis symptoms was correlated with successful cancer treatment, and recurrence of muscle weakness coincided with cancer relapse [17]. Casciola-Rosen et al. found that regenerating cells in myositic muscles and several cancers known to be associated with myositis all express high levels of myositis-specific autoantigens [18]. Therefore, a model of paraneoplasia focusing on the expression of common autoantigens and immuno-targeting between cancer and muscle tissues in myositis has been proposed. The discovery of CAM-associated autoantibodies is important not only for early diagnosis of myositis in patients with greater risk of malignancy, but also for better understanding of the pathogenesis of paraneoplastic myositis.

No attempt has been made to systematically evaluate the association between distinct MSAs and CAM in a large, well-characterized cohort. In this study, we assessed the risk of cancer in patients with different MSAs, and explored the temporal relationship between the onset of myositis and cancer diagnoses, and the correlation between the clinical courses of myositis and cancer in patients with different MSAs. In addition, we also investigated the prognoses of patients with CAM with different MSAs.

## Methods

### Study design

This study involved adult patients with IIMs, from a large cohort of patients diagnosed at China-Japan Friendship Hospital in Beijing, China, between June 2004 and December 2016. This study was approved by the Ethics Committee of the Institutional Review Board at the China-Japan Friendship Hospital, and written informed consent was obtained from all the subjects.

### Patients

Patients who fulfilled definite or probable Bohan and Peter myositis criteria for polymyositis (PM)/DM [19, 20], the European Neuromuscular Centre (ENMC) 2003 criteria through muscle biopsy for IMNM [21], or the Sontheimer criteria for amyopathic dermatomyositis (ADM) [22], were included in the analysis as cases of IIMs. In addition, we separated patients with antisynthetase syndrome (ASS) into a single category by applying criteria proposed by Connors et al. [23]. Patients younger than 16 years, or with overlap syndrome or other types of myositis that have very low incidence in China, such as sIBM, were excluded. Patients with ADM were grouped together with patients with DM. Of the 691 patients identified, 62 patients were excluded due to absence of serum samples. Age-appropriate cancer screening and/or computed tomography of the chest, abdomen, and pelvis were performed in all patients at their initial clinic visit, and the occurrence of malignancy was closely monitored during follow up. All malignancies were confirmed by histopathological findings. Two patients with a suspected yet unconfirmed diagnosis of malignancy were excluded from the analysis to avoid misclassification of cancer status. Therefore, our study population comprised 627 patients. CAM was defined as cancer occurring within 3 years of the disease onset (before or after), and in addition those who had paraneoplastic features [24], even though the interval between the onset of myositis and cancer diagnosis was more than 3 years, were also considered as having CAM.

### Data collecting and recording

Patients’ medical records were reviewed to obtain information about demographic and clinical data and cancer-related data. When patients with IIMs had two or more kinds of malignancy or their recurrence, the time intervals were calculated from the date of the first diagnosis of IIMs to the cancer diagnosis/recurrence immediately after the first diagnosis. A standardized physician questionnaire that captured the clinical features (manual muscle testing, when possible), laboratory data (when possible), the time of cancer diagnosis, type of cancer, the time of death, physician global activity, patients global activity, and therapeutic usage and response was used during follow up in our outpatient clinic, re-hospitalization or telephone interview. In this study, physician global assessment (PGA) using a continuous 10-cm visual analog scale (VAS) was used to rate disease activity. The VAS is a continuous scale where 0 cm represents no disease activity and 10 cm represents extremely severe disease. At each visit the physician compared disease activity to the previous study visit and a 2-cm change in the PGA VAS was regarded as a clinically significant improvement or deterioration [25, 26]. The last follow-up rate was 93.0% as of February 2017, with a median follow-up duration of 33.0 months (IQR 13 to 61 months).

### Assays for myositis-specific antibodies

Serum samples were routinely collected from the patients at initial diagnosis or the follow-up visit, and stored at −80 °C. Serum MSAs including anti-Mi-2α, anti-Mi-2β, anti-TIF1-γ, anti-NXP2, anti-SAE1, anti-MDA5, anti-SRP, anti-Jo-1, anti-PL-7, anti-PL-12, anti-OJ and anti-EJ were detected using commercially available kits (EUROIMMUN, Germany). Anti-HMGCR autoantibodies were evaluated by ELISA (INOVA Diagnostics, USA). The serum sample collected closest to the diagnosis of cancer was examined in this study.

### Statistical analysis

Comparisons of median duration of IIMs at cancer-diagnosis among MSA subgroups were performed using the Kruskal-Wallis test. Fisher’s exact test was employed for comparison of the incidence of cancer among IIM subgroups and comparison of MSA prevalence in different categories of cancers. A *p* value <0.05 was considered significant.

The cancer risk associated with different MSAs was estimated as a standardized incidence ratio (SIR) as previously described [11]. We compared the incidence of cancer over the 6-year period spanning 3 years preceding and 3 years following myositis onset relative to the expected rate of cancer diagnosis in an age-matched and sex-matched population over a 6-year span. Data on the cancer incidence in the general population were obtained from the Chinese Cancer Registry Annual Report of National Central Cancer Registry [27]. Most patient data accounted for 6 person-years around the onset of myositis, except patients who died or were diagnosed with malignancy, who were censored at the time of death or at the time of malignancy diagnosis. Patients with a diagnosis of cancer occurring more than 3 years after the onset of myositis were censored and accounted for 6 person-years. Patients who developed cancer more than 3 years before their onset of myositis were excluded from this study as well. The expected number of cancer cases was obtained by multiplying the patient-years at risk in each 5-year age group by the corresponding sex-specific and age-specific incidence rate. The observed number of cancer incidents was divided by the expected number to obtain the standardized incidence ratio estimate. The 95% confidence intervals (CI) for SIRs were obtained from Poisson distribution tables [28]. Survival was estimated using Kaplan-Meier curves; results were compared using the log-rank test. Age-adjusted and sex-adjusted hazard ratios for mortality were calculated using a Cox hazard regression model. All analyses were performed using SPSS version 17.0 (SPSS, Chicago IL, USA) and Graphpad Prism version 6.00 software (La Jolla, CA, USA).

## Results

### Baseline characteristics of patients

In order to define the exact association between individual types of MSA and cancer, 10 patients with a combination of different types of MSA were excluded, except for the 9 patients who had antibodies to both anti-Mi-2α and anti-Mi-2β (see details in Additional file 1). Thus, 617 patients were finally studied. Of these 617 patients, 72 (11.7%) had malignancies, including 54 of the 378 patients with DM (14.3%), 6 of the 70 patients with PM (8.6%), 10 of the 136 patients with ASS (7.4%) and 2 of the 33 patients with IMNM (6.1%). There was no significant difference in the incidence of malignancy among these IIM subgroups (*p* > 0.05). Characteristics of the entire study population are shown in Table 1. One patient had had two malignancies before and at the time of DM diagnosis: the characteristics of cancer patients are shown in the supporting information in Additional file 1: Table S1. Among these 72 individuals who collectively had 73 malignancies, 38 tested positive for anti-TIF1-γ, 3 for anti-NXP2, 4 for anti-SAE1, 10 for anti-aminoacyl-tRNA-synthetase (anti-ARS) antibodies (including 5 with anti-Jo-1, 3 with anti-PL-12, 1 with anti-PL-7 and 1 with anti-EJ antibodies), 1 each for anti-MDA5, anti-HMGCR, and anti-SRP, and 14 patients were MSAs- negative (patients who carried none of these MSAs, hereinafter referred as the “MSAs-” group). Of the 72 patients with cancer and IIMs, 60 (83.3%) patients were classified as having CAM, including 58 patients who developed cancer within 3 years of the onset of myositis and 2 patients who presented with paraneoplastic features (their cancer emerged with the recurrence of previously diagnosed IIMs, and/or myositis improved after removal of the cancer), although their cancer developed after 4.1 and 5 years, respectively, of the onset of myositis.

**Table 1:** Main characteristics of the entire study population

Characteristics	Patients with IIMs and cancer	Patients with CAM^a^	Patients with IIMs without cancer
Number of patients	72	60	545
Myositis subtype, *n* (%)			
PM	6 (8.3)	3 (5.0)	92 (16.9)
DM	63 (87.5)	56 (93.3)	418 (76.7)
IMNM	3 (4.2)	1 (1.7)	35 (6.4)
Male, *n* (%)	26 (36.1)	24 (40%)	168 (30.8)
Age at myositis onset, mean ± SD years	57.7 ± 14.2	58.0 ± 13.1	47.0 ± 14.1
Age at cancer diagnosis, mean ± SD years	57.5 ± 13.6	58.4 ± 12.9	NA
Time from first symptoms to myositis diagnosis, mean ± SD months (*n*)	12.3 ± 35.6 (72)	7.9 ± 21.9 (60)	22.1 ± 35.1 (512)
Cancer prior to the onset of myositis, mean ± SD, months (*n*)	56.1 ± 91.3 (19)	11.8 ± 13.4 (13)	NA
Cancer after the onset of myositis, mean ± SD, months (*n*)	22.8 ± 42.8 (53)	11.7 ± 12.5 (47)	NA
Follow up, median, IQR, months (*n*)	20.0, 9.0–45.0 (67)	19.0, 9.0–41.8 (55)	34.0,15.0–63.0 (507)

*Abbreviations*: *n* number, *CAM* cancer-associated myositis, *IIMs* idiopathic inflammatory myopathies, *PM* polymyositis, *DM* dermatomyositis, *IMNM* immune-mediated necrotizing myopathy, *SD* standard deviation, *IQR* interquartile range, *NA* not applicable

^a^CAM was defined as cancer occurring within 3 years of the disease onset (before or after), in addition, those who had paraneoplastic features but the interval between the onset of myositis and cancer diagnosis was > 3 years were also considered to have CAM

### Associations between MSAs and cancer risk

To determine whether the risk of cancer was greater within 3 years (either before or after) of myositis onset in patients carrying different MSAs, we compared the patients with age-matched and sex-matched counterparts in the general Chinese population. Overall cancer risk was significantly elevated in anti-TIF1-γ-positive patients (SIR = 17.28, 95% CI 11.94 to 24.14), anti-NXP2-positive patients (SIR = 8.14, 95% CI 1.63 to 23.86), anti-SAE1-positive patients (SIR = 12.92, 95% CI 3.23 to 32.94), and in MSAs- patients (SIR = 3.99, 95% CI 1.96 to 7.14). In contrast, no increased risk was observed in anti-HMGCR-positive patients (SIR = 3.0, 95% CI 0.30 to 16.83), anti-Jo-1-positive patients (SIR = 2.90, 95% CI 0.87 to 12.74), anti-PL-7 positive patients (SIR = 2.90, 95% CI 0.87 to 12.74) and anti-PL-12-positive patients (SIR = 6.29, 95% CI 0.63 to 35.28). Patients with anti-Mi-2, anti-SRP, anti-MDA5, anti-PL-7, anti-EJ, or anti-OJ antibodies did not develop cancer within 3 years of myositis onset (Table 2).

**Table 2:** Risk of cancer within 3 years of myositis onset with different MSAs compared to the general population

Autoantibodies	Total^a^	Observed^b^	Expected^c^	SIR (95% CI)
Anti-TIF1-γ	89	34	1.97	17.28 (11.94, 24.14)
Anti-NXP2	42	3	0.37	8.14 (1.63, 23.86)
Anti-SAE1	13	4	0.31	12.92 (3.23, 32.94)
Anti-Mi-2	24	0	0.53	0 (0, 7.03)
Anti-HMGCR	21	1	0.33	3.0 (0.30, 16.83)
Anti-SRP	30	0	0.32	0 (0, 11.47)
Anti-MDA5	92	0	1.25	0 (0, 2.97)
Anti-Jo-1	63	3	1.01	2.98 (0.60, 8.74)
Anti-PL-7	33	0	0.71	0 (0, 5.24)
Anti-PL-12	17	2	0.34	5.92 (0.59, 21.31)
Anti-EJ	22	0	0.40	0 (0, 9.36)
Anti-OJ	1	0	0.06	0 (0, 58.78)
MSAs-^d^	170	11	2.76	3.99 (1.96, 7.14)

*Abbreviations*: *SIR* standardized incidence ratio, *MSAs* myositis specific autoantibodies

^a^Number of autoantibody-positive patients

^b^Observed cancer cases within 3 years of myositis onset

^c^Expected cancer cases based on estimates in the general population in China, after adjustment for age and gender

^d^Patients who were negative for all of the MSAs listed

### Associations between MSAs and cancer type in patients with CAM

**Fig. 1:**
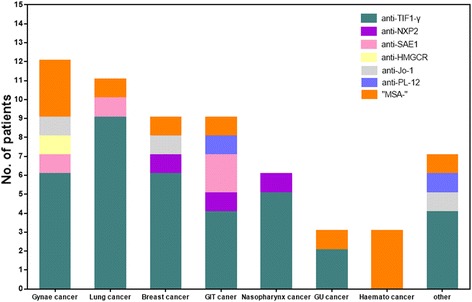
Frequency of each myositis-specific autoantibody (MSA) subtype in subgroups of different types of cancer. Gynae, Gynaecological; GIT, Gastrointestinal; GU, Genitourinary; Haemato, Haematological. Other includes thyroid, liver, thymus, larynx and salivary duct cancer

Anti-TIF1-γ antibodies, anti-NXP2 antibodies, anti-SAE1 antibodies, anti-HMGCR antibodies, anti-Jo-1 antibodies, anti-PL-12 antibodies, and MSAs- were identified in 36, 3, 4, 1, 3, 2 and 11 of the 60 patients with CAM, respectively. Detailed analysis of cancer subtypes in patients with CAM was performed in relation to the subgroups (including six MSA subtypes and one MSAs- group) (Fig. 1).

In our cohort, anti-TIF1-γ antibodies occurred most frequently together with each subtype of cancer, except for hematological cancers. Anti-NXP2 antibodies were detected in three patients: one with breast cancer, one with gastric cancer, and one with nasopharyngeal cancer. Patients in the group carrying anti-SAE1 antibodies also had cervical, lung, esophageal and rectal cancers. MSAs- patients mainly had hematological or gynaecological cancer. As shown in Fig. 1, no MSA was found to be associated with a certain particular type of malignancy (*p* = 0.36).

### Survival in patients with CAM within MSA subgroups

**Fig. 2:**
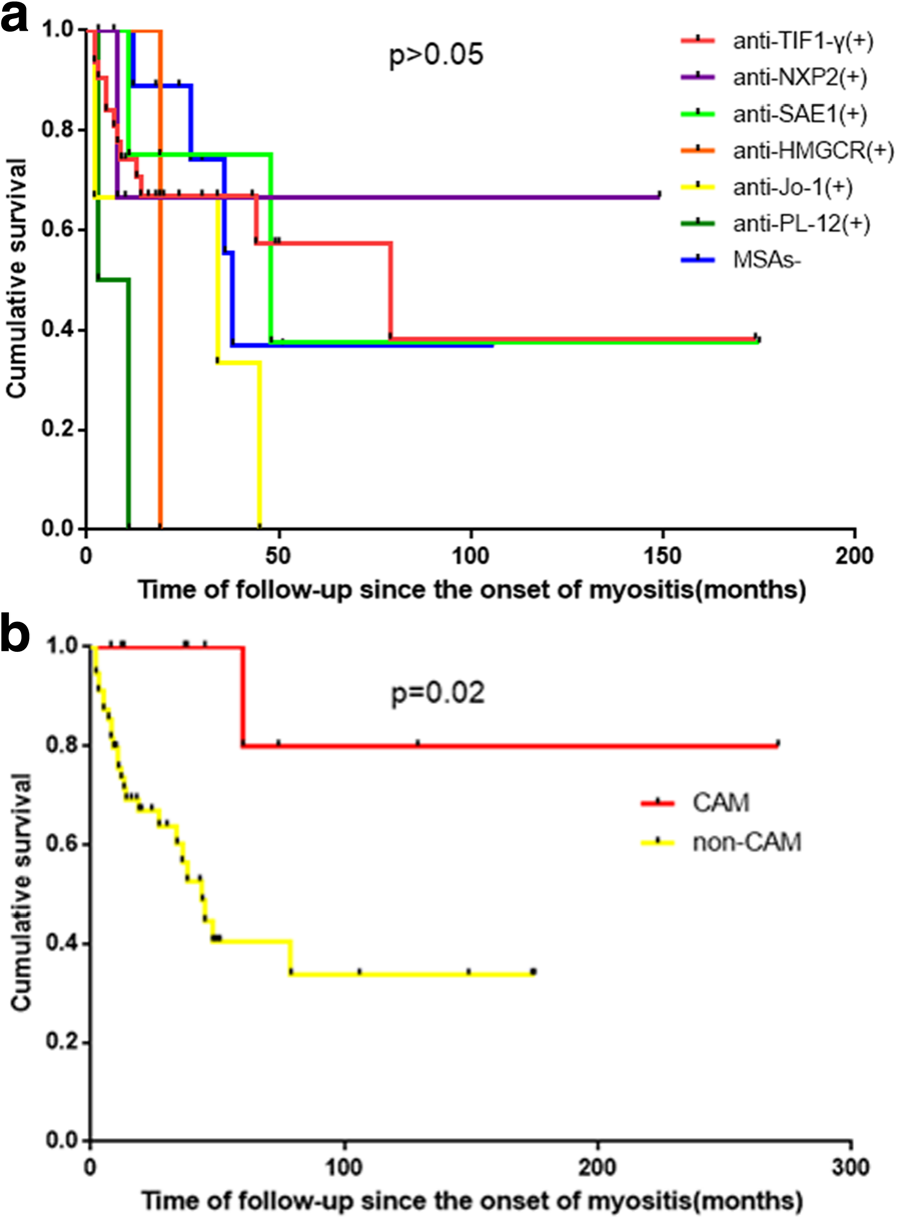
Survival analysis: Kaplan-Meier method with log-rank test for comparison of survival curves. **a** Patients with cancer-associated myositis (CAM) are compared based on the serological status. **b** Survival analysis in patients with CAM and those with cancer unrelated to myositis. Survival was not significantly different depending on the presence of myositis-specific autoantibodies (MSAs) in patients with CAM (*p* > 0.05). Survival in patients with CAM was shorter than in patients with cancer unrelated to myositis (*p* = 0.02 by log-rank test)

Of the 60 patients with CAM, 4 were lost to followup. At the last follow up, 55.4% of patients with CAM (31 out of the 56) were alive. The median follow-up time, measured from the time of myositis onset, was 24.0 months (IQR 16.0 to 49.0 months). Other patients with CAM died from progression of cancer, infection, or severe myositis symptoms such as respiratory failure, with median survival time of 11.0 months (IQR 5.0 to 35.0 months). Comparing prognoses between patients with CAM with different MSAs, there were no significant differences between survival rates in the different MSA subgroups (logrank *p* > 0.05) (Fig. 2a). Further investigation on whether the prognosis of patients with IIMs with cancer was different in those with CAM and those with cancer unrelated to myositis showed that patients with myositis that was unrelated to cancer survived longer than patients with CAM. According to Kaplan-Meier analysis, the mean survival time was 77.3 months (95% CI 51.8 to 102.9) in CAM vs 222.8 months (95% CI 154.8 to 302.8) in myositis unrelated to cancer (logrank *p* = 0.02) (Fig. 2b).The age-adjusted and sex-adjusted hazard ratio for mortality in CAM vs cancer unrelated to myositis was 10.8 (95% CI 1.38 to 84.5, *p* = 0.02).

### Temporal relationship between the onset of myositis and cancer diagnosis within MSA subgroups

**Fig. 3:**
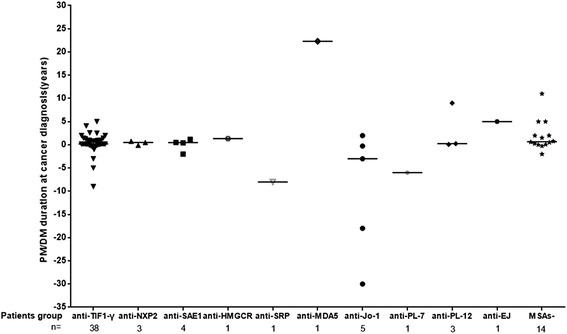
Distribution of idiopathic inflammatory myopathy (IIM) duration at cancer diagnosis by autoantibody status. There were tight temporal relationships between the onset of myositis and cancer diagnosis in patients with antibodies to TIF1-γ, NXP2, and SAE1. The groups of patients with anti-HMGCR, anti-Jo-1 antibodies, anti-PL-12 antibodies, and MSAs-negative patients had similar close temporal relationships between the onset of myositis and cancer diagnosis. Horizontal bars indicate median duration). PM, polymyositis; DM, dermatomyositis

A previous study demonstrated a close temporal relationship between myositis onset and cancer diagnosis in patients carrying anti-TIF1-γ antibodies [29]. Therefore, we wondered whether there is such a relationship in other MSA subgroups. We examined the cancer-IIM interval in each MSA subgroup and found that the median duration of IIMs at cancer-diagnosis did not differ significantly between the groups: +0.19 years in the anti-TIF1-γ group (IQR −0.02 to +0.19 years) (the plus sign signifies cancer developing after myositis onset and the minus sign signifies cancer developing before myositis); +0.5 years in the anti-NXP2 group (IQR −0.08 to +0.75 years); +0.46 years in the anti-SAE1 group (IQR −1.4 to +1.0 years); −3.00 years in the anti-Jo-1 group (IQR −24.0 to +0.88 years); +0.25 years in the anti-PL-12 group (IQR +0.08 to +9.0 years); and +0.67 years in the MSAs- group (IQR +0.06 to +2.75 years) (*p* > 0.05). One patient each from the anti-HMGCR, anti-SRP, anti-PL-7, anti-EJ, and anti-MDA5 groups had cancer-IIM intervals of 1.33, 8.0, 6.0, 5.0, and 22.33 years, respectively (Fig. 3).

### Clinical course of myositis and cancer within MSA subgroups

Of the 72 patients who had IIMs and cancer, 5 were lost to follow up. Antineoplastic treatment was administered to 65 patients, and 2 patients were not treated or received only symptomatic treatment because of their poor clinical condition. A parallel between the clinical course of myositis and cancer was observed in patients carrying anti-TIF1, anti-NXP2, anti-SAE1, anti-Jo-1, anti-PL-12, and anti-HMGCR antibodies, and in the MSAs- patients (Table 3).

**Table 3:** The correlation between clinical course of myositis and cancer

Autoantibodies	Patients with paralleled clinical course, number^a^	Patients with non-paralleled clinical course, number	Total number^b^
Anti-TIF1-γ	20	14	34
Anti-NXP2	2	1	3
Anti-SAE1	2	2	4
Anti-HMGCR	1	0	1
Anti-SRP	0	1	1
Anti-Jo-1	1	4	5
Anti-PL-7	0	1	1
Anti-PL-12	1	2	3
Anti-EJ	0	1	1
Anti-MDA5	0	1	1
MSAs-^c^	3	10	13

*Abbreviations*: *MSAs* myositis specific autoantibodies

^a^A paralleled clinical course meant that myositis emerged/deteriorated with the progression/recurrence of previously diagnosed cancers, cancer emerged with the recurrence of previously diagnosed IIMs, and/or myositis improved after removal or effective treatment of the cancer

^b^Four patients with anti-TIF1-γ and one MSA-negative patient were lost to follow up

^c^Negative for all of the listed MSAs

## Discussion

The present study is the first attempt to systematically explore whether other MSAs are also associated with CAM in addition to anti-TIF1-γ antibodies. The discovery of such an association may hint at whether cancer could be a general trigger of autoimmunity in patients with IIM. In this large longitudinal cohort study, we demonstrated that patients with IIM who carry anti-TIF1-γ, anti-NXP2, or anti-SAE1 antibodies, and those who are MSAs-negative have increased risk of cancer compared to age-matched and sex-matched control subjects. Thus, these patients especially should undergo comprehensive cancer screening.

The theory proposed by Casciola-Rosen et al. may provide insight into paraneoplastic associations with CAM. A case report from Japan describes a patient carrying anti-TIF1-γ who had DM and endometrial cancer, and whose tumor cells expressed high levels of TIF1-γ. This finding led to the hypothesis that TIF1-γ was overexpressed in tumor cells, induced the formation of autoantibodies, and caused paraneoplastic DM in this patient [30]. Correspondingly, overexpressed TIF1-γ has been identified in the regenerating fibers of patients with DM [31]. These findings could prompt research into other non-TIF1-γ antigens that might be involved in the pathogenesis of cancer and autoimmunity. In the current study, we found that anti-NXP2 and anti-SAE1 are also associated with an increased risk of cancer, in addition to anti-TIF1-γ. It is interesting that their corresponding autoantigens are all involved in the SUMO pathway [32–34], which plays an important role in regulating multiple cellular processes [35]. Aberrant SUMOylation may thus play a fundamental role in both IIMs and cancer [35].

Our study showed a close temporal relationship between the onset of myositis and cancer diagnosis not only in the anti-TIF1-γ group, but also in the other MSA groups. In addition, a parallel between the clinical course of myositis and cancer was observed in patients from multiple MSA subgroups. These results strongly suggest that besides anti-TIF1-γ, patients with IIMs who carry other MSAs might experience a paraneoplastic syndrome in which the immune system is responding to cancer. In addition to patients with IIMs with autoantibodies to TIF1-γ, NXP2 and SAE1, we also noted that in some cases in the anti-ARS group (anti-Jo-1 and anti-PL-12) there was temporally proximate occurrence of the cancer and myositis onset. Moreover, a parallel between the clinical course of myositis and cancer was observed in patients carrying anti-Jo-1 and anti-PL-12. Similarly, in several studies, paraneoplastic myositis has been observed in patients with IIMs carrying anti-ARS antibodies [6–9, 36, 37]. Of particular interest is that multiple members of the aminoacyl-tRNA-synthetase family play important roles in malignant processes [38]. These data suggest that in some cases, anti-ARS antibody production might also be driven by malignancy. Interestingly, we found one of the patients with CAM was anti-HMGCR-positive, and this observation was also reported in other studies [11, 12, 39]. Considering the overexpression of HMGCR in cancer tissue and regenerating muscle cells [40, 41], together with the finding that the target of this antibody plays a role in oncogenesis [42–44], an association with cancer may have contributed to the development of anti-HMGCR-positive myopathy.

As we know, paraneoplastic neurological disorder (PND) is a typical paraneoplastic disorder. Among patients with PND, each specific serum antibody is associated with a narrow spectrum of clinical syndromes and a restricted subgroup of cancers. Unlike PND, we did not find such a close association in patients with CAM. Anti-TIF1-γ antibodies, the antibodies that associate most strongly with cancer, had been detected among various subtypes of cancer, except for hematological cancers. MSAs-negative patients, however, mainly had hematological cancers, suggesting that more attention should be given to the screening of hematological cancers in IIM patients who are negative for all of these MSAs. We found that MSAs-negative patients with IIM faced an increased risk of cancer with the etiological factors remaining speculative. However, a possible explanation may also be underlying autoimmunity, therefore, identifying the currently unknown autoantigens in the MSAs-negative group is an important priority.

It is worth noting that patients classified as having CAM had poorer prognoses compared to those whose cancer thought to be unrelated to myositis. Similar results were recently reported by Kang et al. [45]. Possible explanations are that prognosis and life expectancy in patients with cancer are determined by the underlying malignant disease [46], and the most common cancer subtype ihe n CAM subgroup was lung cancer, especially small-cell lung cancer, which is well-known for its poor prognosis. In addition, the advanced cancers might have increased immunogenicity based on the disease duration and the quantity of tumor antigens that had been exposed to the immune system. Therefore, cancers that are temporally proximate to myositis onset may be mostly advanced cancers that are more likely to cause autoimmunity.

This study had several limitations. First, although this is the largest cohort study published to date to systematically evaluate the association between distinct MSAs and cancer in patients with IIMs, inclusion of patients from a single tertiary referral center may make it subject to possible biases. Second, the relatively small sample of patients with cancer makes it very difficult to draw firm conclusion on whether the prognoses differed significantly in patients with different MSAs. Third, the rigorous cancer screening at diagnosis in patients with IIMs might have led to overestimation of cancer risk when calculating the SIRs, though the schemes for cancer screening were the same in each MSAs subgroup. Fourth, we used the line blot (LB) and ELISA methods to detect the MSAs, and did not perform immunoprecipitation (IP)-western blotting (WB) assays to further confirm the specificities of these antibodies. The possible false positive or negative results might have led to potential for bias. Fifth, combination of our results with other demographic, clinical and laboratory risk factors will promote our understanding of the distinction between CAM and pure IIMs. Furthermore, three patients with CAM carrying anti-Mi-2β and other kinds of antibodies were excluded from the cohort. Of note, Hengstman et al. reported that anti-Mi-2β antibodies against the N-terminal fragment are associated with increased risk of cancer, and Mi-2 has been found to be involved in mediating SUMO-dependent repression [47]. Thus, further studies should be done to confirm whether anti-Mi-2 is associated with cancer in patients with IIMs. Finally, in some cases, we lacked serum samples taken at malignancy onset. To address this, we evaluated the serum sample taken closest to cancer diagnosis; the median time interval between cancer diagnosis and serum sampling was 7 months.

## Conclusions

Our study showed that multiple MSAs are associated with cancer in IIMs. Further studies are necessary to evaluate the biological link between these specific autoantibodies and malignancy in IIMs. Definition of antigenic epitopes, identification of MSA-specific T cells capable of lysing tumor cells, and examination of IIM-antigen expression, mutation, or modification in target tissues – such as skin, muscle, and tumor tissues – may provide new insights into the paraneoplastic hypothesis of myositis. Investigation of paraneoplastic myositis will provide unique insights into the mechanisms of tumor immunity and the opportunity to apply this knowledge to patients with cancer in general.

## Additional file

Additional file 1: The final population studied. **Table S1.** Characteristics of patients with IIMs and cancer. (DOCX 20 kb)

## Abbreviations

ADM: Amyopathic dermatomyositis
anti-ARS: Anti-aminoacyl-tRNA-synthetase
ASS: Antisynthetase syndrome
CAM: Cancer-associated myositis
CI: Confidence intervals
DM: Dermatomyositis
ELISA: Enzyme-linked immunosorbent assay
ENMC: European Neuromuscular Centre
IIMs: Idiopathic inflammatory myopathies
IMNM: Immune-mediated necrotizing myopathy
MSAs: Myositis-specific autoantibodies
PM: Polymyositis
PND: Paraneoplastic neurological disorders
sIBM: Sporadic inclusion body myositis
SIR: Standardized incidence ratio

## References

[1] Lu X, Peng Q, Wang G (2015). Discovery of new biomarkers of idiopathic inflammatory myopathy. Clin Chim Acta..

[2] Troyanov Y, Targoff IN, Tremblay JL, Goulet JR, Raymond Y, Senecal JL (2005). Novel classification of idiopathic inflammatory myopathies based on overlap syndrome features and autoantibodies: analysis of 100 French Canadian patients. Medicine (Baltimore).

[3] Ichimura Y, Matsushita T, Hamaguchi Y, Kaji K, Hasegawa M, Tanino Y (2012). Anti-NXP2 autoantibodies in adult patients with idiopathic inflammatory myopathies: possible association with malignancy. Ann Rheum Dis..

[4] Fiorentino DF, Chung LS, Christopher-Stine L, Zaba L, Li S, Mammen AL (2013). Most patients with cancer-associated dermatomyositis have antibodies to nuclear matrix protein NXP-2 or transcription intermediary factor 1gamma. Arthritis Rheum..

[5] Albayda J, Pinal-Fernandez I, Huang W, Parks C, Paik J, Casciola-Rosen L, et al. Dermatomyositis patients with anti-nuclear matrix protein-2 autoantibodies have more edema, more severe muscle disease, and increased malignancy risk. Arthritis Care Res. 2017;69:1771-6. 10.1002/acr.23188 PMC5509530 28085235

[6] Legault D, McDermott J, Crous-Tsanaclis AM, Boire G (2008). Cancer-associated myositis in the presence of anti-Jo1 autoantibodies and the antisynthetase syndrome. J Rheumatol..

[7] Marie I, Hatron PY, Cherin P, Hachulla E, Diot E, Vittecoq O (2013). Functional outcome and prognostic factors in anti-Jo1 patients with antisynthetase syndrome. Arthritis Res Ther..

[8] Marie I, Josse S, Decaux O, Dominique S, Diot E, Landron C (2012). Comparison of long-term outcome between anti-Jo1- and anti-PL7/PL12 positive patients with antisynthetase syndrome. Autoimmun Rev..

[9] Lega JC, Fabien N, Reynaud Q, Durieu I, Durupt S, Dutertre M (2014). The clinical phenotype associated with myositis-specific and associated autoantibodies: a meta-analysis revisiting the so-called antisynthetase syndrome. Autoimmun Rev..

[10] Hengstman GJ, Vree Egberts WT, Seelig HP, Lundberg IE, Moutsopoulos HM, Doria A (2006). Clinical characteristics of patients with myositis and autoantibodies to different fragments of the Mi-2 beta antigen. Ann Rheum Dis..

[11] Allenbach Y, Keraen J, Bouvier AM, Jooste V, Champtiaux N, Hervier B (2016). High risk of cancer in autoimmune necrotizing myopathies: usefulness of myositis specific antibody. Brain..

[12] Kadoya M, Hida A, Hashimoto Maeda M, Taira K, Ikenaga C, Uchio N (2016). Cancer association as a risk factor for anti-HMGCR antibody-positive myopathy. Neurol Neuroimmunol Neuroinflamm..

[13] Ceribelli A, Fredi M, Taraborelli M, Cavazzana I, Franceschini F, Quinzanini M (2012). Anti-MJ/NXP-2 autoantibody specificity in a cohort of adult Italian patients with polymyositis/dermatomyositis. Arthritis Res Ther..

[14] Brouwer R, Hengstman GJ, Vree Egberts W, Ehrfeld H, Bozic B, Ghirardello A (2001). Autoantibody profiles in the sera of European patients with myositis. Ann Rheum Dis..

[15] Love LA, Leff RL, Fraser DD, Targoff IN, Dalakas M, Plotz PH (1991). A new approach to the classification of idiopathic inflammatory myopathy: myositis-specific autoantibodies define useful homogeneous patient groups. Medicine (Baltimore).

[16] O'Hanlon TP, Carrick DM, Targoff IN, Arnett FC, Reveille JD, Carrington M (2006). Immunogenetic risk and protective factors for the idiopathic inflammatory myopathies: distinct HLA-A, -B, -Cw, -DRB1, and -DQA1 allelic profiles distinguish European American patients with different myositis autoantibodies. Medicine (Baltimore).

[17] Danko K, Ponyi A, Molnar AP, Andras C, Constantin T (2009). Paraneoplastic myopathy. Curr Opin Rheumatol..

[18] Casciola-Rosen L, Nagaraju K, Plotz P, Wang K, Levine S, Gabrielson E (2005). Enhanced autoantigen expression in regenerating muscle cells in idiopathic inflammatory myopathy. J Exp Med..

[19] Bohan A, Peter JB (1975). Polymyositis and dermatomyositis (first of two parts). N Engl J Med..

[20] Bohan A, Peter JB (1975). Polymyositis and dermatomyositis (second of two parts). N Engl J Med..

[21] Hoogendijk JE, Amato AA, Lecky BR, Choy EH, Lundberg IE, Rose MR (2004). 119th ENMC international workshop: trial design in adult idiopathic inflammatory myopathies, with the exception of inclusion body myositis, 10-12 October 2003, Naarden, The Netherlands. Neuromuscul Disord..

[22] Sontheimer RD (2002). Would a new name hasten the acceptance of amyopathic dermatomyositis (dermatomyositis sine myositis) as a distinctive subset within the idiopathic inflammatory dermatomyopathies spectrum of clinical illness?. J Am Acad Dermatol..

[23] Connors GR, Christopher-Stine L, Oddis CV, Danoff SK (2010). Interstitial lung disease associated with the idiopathic inflammatory myopathies: what progress has been made in the past 35 years?. Chest..

[24] Azar L, Khasnis A (2013). Paraneoplastic rheumatologic syndromes. Curr Opin Rheumatol..

[25] Anyanwu CO, Fiorentino DF, Chung L, Dzuong C, Wang Y, Okawa J (2015). Validation of the Cutaneous Dermatomyositis Disease Area and Severity Index: characterizing disease severity and assessing responsiveness to clinical change. Br J Dermatol..

[26] Farrar JT, Berlin JA, Strom BL (2003). Clinically important changes in acute pain outcome measures: a validation study. J Pain Symptom Manag..

[27] Chen W, Zheng R, Zeng H, Zhang S, He J (2015). Annual report on status of cancer in China, 2011. Chin J Cancer Res..

[28] Diem K, Seldrup J, Lentner C (1982). Medical Education Division. Poisson distribution of 95% confidence limits. Geigy scientific tables.

[29] Hida A, Yamashita T, Hosono Y, Inoue M, Kaida K, Kadoya M (2016). Anti-TIF1-gamma antibody and cancer-associated myositis: a clinicohistopathologic study. Neurology..

[30] Kasuya A, Hamaguchi Y, Fujimoto M, Tokura Y (2013). TIF1gamma-overexpressing, highly progressive endometrial carcinoma in a patient with dermato-myositis positive for malignancy-associated anti-p155/140 autoantibody. Acta Derm Venereol..

[31] Mohassel P, Rosen P, Casciola-Rosen L, Pak K, Mammen AL (2015). Expression of the dermatomyositis autoantigen transcription intermediary factor 1gamma in regenerating muscle. Arthritis Rheumatol..

[32] Ishikawa A, Muro Y, Sugiura K, Akiyama M (2012). Development of an ELISA for detection of autoantibodies to nuclear matrix protein 2. Rheumatology (Oxford).

[33] Fiorentino D, Casciola-Rosen L (2012). Autoantibodies to transcription intermediary factor 1 in dermatomyositis shed insight into the cancer-myositis connection. Arthritis Rheum..

[34] Fattet L, Ay AS, Bonneau B, Jallades L, Mikaelian I, Treilleux I (2013). TIF1gamma requires sumoylation to exert its repressive activity on TGFbeta signaling. J Cell Sci..

[35] Mattoscio D, Chiocca S (2015). SUMO pathway components as possible cancer biomarkers. Futur Oncol (London, England).

[36] Rozelle A, Trieu S, Chung L (2008). Malignancy in the setting of the anti-synthetase syndrome. J Clin Rheumatol..

[37] Chinoy H, Fertig N, Oddis CV, Ollier WE, Cooper RG (2007). The diagnostic utility of myositis autoantibody testing for predicting the risk of cancer-associated myositis. Ann Rheum Dis..

[38] Park SG, Schimmel P, Kim S (2008). Aminoacyl tRNA synthetases and their connections to disease. Proc Natl Acad Sci USA.

[39] Mizuma A, Kouchi M, Netsu S, Yutani S, Kitao R, Suzuki S (2017). Paraneoplastic anti-3-hydroxy-3-methylglutary-coenzyme A reductase antibody-positive immune-mediated necrotizing myopathy in a patient with uterine cancer. Intern Med (Tokyo, Japan).

[40] Gustbee E, Tryggvadottir H, Markkula A, Simonsson M, Nodin B, Jirstrom K (2015). Tumor-specific expression of HMG-CoA reductase in a population-based cohort of breast cancer patients. BMC Clin Pathol..

[41] Mammen AL, Chung T, Christopher-Stine L, Rosen P, Rosen A, Doering KR (2011). Autoantibodies against 3-hydroxy-3-methylglutaryl-coenzyme A reductase in patients with statin-associated autoimmune myopathy. Arthritis Rheum..

[42] Clendening JW, Pandyra A, Boutros PC, Ghamrasni SE, Khosravi F, Trentin GA (2010). Dysregulation of the mevalonate pathway promotes transformation. Proc Natl Acad Sci USA.

[43] Qiu Z, Yuan W, Chen T, Zhou C, Liu C, Huang Y (2016). HMGCR positively regulated the growth and migration of glioblastoma cells. Gene..

[44] Chushi L, Wei W, Kangkang X, Yongzeng F, Ning X, Xiaolei C (2016). HMGCR is up-regulated in gastric cancer and promotes the growth and migration of the cancer cells. Gene..

[45] Kang EH, Lee SJ, Ascherman DP, Lee YJ, Lee EY, Lee EB (2016). Temporal relationship between cancer and myositis identifies two distinctive subgroups of cancers: impact on cancer risk and survival in patients with myositis. Rheumatology (Oxford).

[46] Ponyi A, Constantin T, Garami M, Andras C, Tallai B, Vancsa A (2005). Cancer-associated myositis: clinical features and prognostic signs. Ann NY Acad Sci..

[47] Stielow B, Sapetschnig A, Kruger I, Kunert N, Brehm A, Boutros M (2008). Identification of SUMO-dependent chromatin-associated transcriptional repression components by a genome-wide RNAi screen. Mol Cell..

