# Correction to: identification of multiple cancer-associated myositis-specific autoantibodies in idiopathic inflammatory myopathies: a large longitudinal cohort study

**DOI:** 10.1186/s13075-018-1549-4

**Published:** 2018-04-11

**Authors:** Hanbo Yang, Qinglin Peng, Liguo Yin, Shanshan Li, Jingli Shi, Yamei Zhang, Xin Lu, Xiaoming Shu, Sigong Zhang, Guochun Wang

**Affiliations:** 1 Department of Rheumatology, Beijing Key Lab for Immune-Mediated Inflammatory Diseases, China-Japan Friendship Hospital, 2 Yinhua Road, Chaoyang District, Beijing, 100029 China; 2 Graduate School of Peking Union Medical College, Beijing, 100730 China; 3 Department of Rheumatology, Lanzhou University Second Hospital, Gansu province, 730046 China

## Correction

After publication of the article [1], it has been brought to our attention that the labels in Fig. 2b have been switched and are as a result incorrect. The label for the red line should have the label “non-CAM” and the yellow line “CAM”.
