# Supplementary material for: Identification of multiple cancer-associated myositis-specific autoantibodies in idiopathic inflammatory myopathies: a large longitudinal cohort study
Source: Arthritis Res Ther. 2017 Nov 25;19:259. doi: 10.1186/s13075-017-1469-8 (PMC5702134; doi:10.1186/s13075-017-1469-8)
Supplement: Additional file 1: — The final population studied. Table S1. Characteristics of patients with IIMs and cancer. (DOCX 20 kb) [file 13075_2017_1469_MOESM1_ESM.docx]

**Additional file 1**

**The final studied population**

Isolated anti-Mi-2 (included anti-Mi-2α and anti-Mi-2β), anti-ARS antibodies (included anti-Jo-1, anti-PL-7, anti-PL12, anti-EJ, anti-OJ), anti-NXP2 antibodies, anti-TIF1-γ antibodies, anti-MDA5 antibodies, anti-SAE1 antibodies, anti-SRP antibodies and anti-HMGCR antibodies were found in 15, 136, 42, 89, 92, 13, 30, 21 of them, respectively, whereas 19 sera were positive for combinations of antibodies (nine with anti-Mi-2α and anti-Mi-2β; two with anti-Jo-1 and anti-PL-7; two with anti-TIF1-γ and anti-Mi-2β; one with anti-SAE1 and anti-Jo-1; one with anti-Mi-2β and anti-Jo-1, and four sera of patients with cancer, included two with anti-Mi-2β and anti-TIF1-γ; one with anti-Mi-2β and anti-PL-12; one with anti-PL-7 and anti-MDA5) and 170 were negative for all these specificities. Except for the patients who had antibodies to both anti-Mi-2α and anti-Mi-2β, these latter six groups of patients were excluded from further analysis to avoid the confounding effect of double specificities. Thus, 617 patients with isolated MSAs were studied.

**Table S1** Characteristics of patients with IIMs and cancer

| Patients | Diagnosis | Type of cancer | Time from cancer to myositis onset (month)^a^ | Autoantibodies detected | Survival |
| --- | --- | --- | --- | --- | --- |
| 1 | DM | Breast | -12 | TIF1-γ | NA |
| 2 | DM | Lung | +1 | TIF1-γ | Dead |
| 3 | DM | Lung | +17 | TIF1-γ | Dead |
| 4 | DM | Lung | +2.5 | TIF1-γ | Dead |
| 5 | DM | Lung | +2 | TIF1-γ | Dead |
| 6 | DM | Liver | +15.5 | TIF1-γ | Dead |
| 7 | DM | Prostate | +31 | TIF1-γ | NA |
| 8 | DM | Thyroid | -108 | TIF1-γ | Alive |
| 9 | DM | Ovary | +2 | TIF1-γ | Alive |
| 10 | DM | Thyroid | +24 | TIF1-γ | Alive |
| 11 | DM | Breast | +60 | TIF1-γ | Alive |
| 12 | DM | Ovary | +11 | TIF1-γ | NA |
| 13 | ADM | Lung | +49 | TIF1-γ | Dead |
| 14 | DM | Lung | +1 | TIF1-γ | Alive |
| 15 | DM | Nasopharynx | +24 | TIF1-γ | Alive |
| 16 | DM | Nasopharynx | +12 | TIF1-γ | Alive |
| 17 | ADM | Nasopharynx | +10 | TIF1-γ | Alive |
| 18 | DM | Ovary | -3 | TIF1-γ | Alive |
| 19 | DM | Lung | +2 | TIF1-γ | Dead |
| 20 | DM | Ovary | +2 | TIF1-γ | Alive |
| 21 | DM | Breast | -4 | TIF1-γ | Alive |
| 22 | DM | Liver, Ureter | -1 | TIF1-γ | Dead |
| 23 | DM | Thymus | +12 | TIF1-γ | Alive |
| 24 | DM | Breast | -6 | TIF1-γ | Alive |
| 25 | DM | Colon | +2 | TIF1-γ | NA |
| 26 | DM | Lung | +2 | TIF1-γ | Dead |
| 27 | DM | Nasopharynx | +12 | TIF1-γ | Alive |
| 28 | DM | Rectum | -60 | TIF1-γ | Alive |
| 29 | DM | Ovary | -36 | TIF1-γ | Dead |
| 30 | DM | Breast | +12 | TIF1-γ | Alive |
| 31 | DM | Stomach | +8 | TIF1-γ | Dead |
| 32 | DM | Lung | +10 | TIF1-γ | Dead |
| 33 | DM | Ovary | -1 | TIF1-γ | Alive |
| 34 | DM | Nasopharynx | +30 | TIF1-γ | Alive |
| 35 | DM | Esophagus | +1 | TIF1-γ | Alive |
| 36 | DM | Ovary | Same time | TIF1-γ | Alive |
| 37 | DM | Colon | +3 | TIF1-γ | Alive |
| 38 | DM | Breast | +17 | TIF1-γ | Alive |
| 39 | DM | Stomach | -1 | NXP2 | Dead |
| 40 | ADM | Nasopharynx | +6 | NXP2 | Alive |
| 41 | PM | Breast | +9 | NXP2 | Alive |
| 42 | DM | Lung | +5 | SAE1 | Dead |
| 43 | DM | Cervix | -24 | SAE1 | Alive |
| 44 | DM | Esophagus | +14 | SAE1 | Dead |
| 45 | DM | Rectum | +6 | SAE1 | Alive |
| 46 | ASS | Breast | -216 | Jo-1 | Alive |
| 47 | ASS | Thyroid | -360 | Jo-1 | Alive |
| 48 | ASS | Salivary duct | -36 | Jo-1 | Dead |
| 49 | ASS | Breast | +24 | Jo-1 | Dead |
| 50 | ASS | Cervix | -3 | Jo-1 | Dead |
| 51 | ASS | Thyroid | +108 | PL-12 | Alive |
| 52 | ASS | Stomach | +3 | PL-12 | Dead |
| 53 | ASS | Thymus | +1 | PL-12 | Dead |
| 54 | DM | Lymphoma | +24 | MSA- | Dead |
| 55 | DM | Esophagus | +1 | MSA- | Dead |
| 56 | DM | Lymphoma | +6 | MSA- | Alive |
| 57 | DM | Thyroid | +7 | MSA- | Alive |
| 58 | DM | Lymphoma | +18 | MSA- | Dead |
| 59 | ADM | Chorion | -3 | MSA- | Alive |
| 60 | DM | Lung | -24 | MSA- | Alive |
| 61 | DM | Invasive mole | +9 | MSA- | Alive |
| 62 | DM | Leukemia (CLL) | +132 | MSA- | NA |
| 63 | PM | Ovary | +3 | MSA- | Dead |
| 64 | PM | Breast | +24 | MSA- | Alive |
| 65 | PM | Pancreas | +60 | MSA- | Dead |
| 66 | PM | Prostate | Same time | MSA- | Alive |
| 67 | PM | Colon | +60 | MSA- | Alive |
| 68 | ASS | Thyroid | +60 | EJ | Alive |
| 69 | ASS | Breast | -72 | PL-7 | Alive |
| 70 | DM | Larynx | +268 | MDA5 | Alive |
| 71 | IMNM | Cervix | +16 | HMGCR | Dead |
| 72 | IMNM | Bladder | -96 | SRP | Alive |

Abbreviations: PM, polymyositis; DM, dermatomyositis; ADM, amyopathic dermatomyositis; IMNM, immune-mediated necrotizing myopathy; ASS, antisynthetase syndrome; MSA-, negative for all of these MSAs; NA, not available;

a a plus sign means cancer developing after the onset of myositis; conversely, a minus sign means cancer developing before the onset of myositis.
